# Triple negative breast cancer – prognostic factors and survival

**DOI:** 10.2478/v10019-010-0054-4

**Published:** 2010-12-31

**Authors:** Tanja Ovcaricek, Snjezana Grazio Frkovic, Erika Matos, Barbara Mozina, Simona Borstnar

**Affiliations:** Institute of Oncology Ljubljana, Ljubljana, Slovenia

**Keywords:** triple negative breast cancer, prognostic factors, treatment outcome

## Abstract

**Background:**

Triple negative breast cancer (TNBC) is defined by a lack of expression of both estrogen (ER) and progesteron (PgR) receptors as well as human epidermal growth factor receptor 2 (HER2). Our retrospective analysis addressed prognostic factors for short- and long-term outcomes of patients (pts) with TNBC pts treated in routine clinical practice.

**Patient and methods.:**

Our retrospective study included 269 TNBC treated at Institute of Oncology Ljubljana between March 2000 and December 2006. The collected data included patients’, tumours’ and treatments’ characteristics. The survival analyses were performed using the Kaplan-Meier method. The Cox proportional hazard model was used in the multivariate analysis.

**Results:**

The median age of our patients was 55.3 yrs (23–88.5) and the median follow-up was 5.9 yrs (0.3–9.6). Six (2%) pts experienced local only, 79 (92%) pts distal recurrence and 66 (24%) died. The predominant localisation of the first relapse was in visceral organs (70.4%). The 5-year disease-free survival (DFS) for the entire group was 68.2% and the 5-year overall survival (OS) was 74.5%. We found a pattern of high recurrence rate in the first 3 years following the diagnosis and a clear decline in recurrence rate over the next 3 years. In the univariate analysis age, nodal status, size and lymphovascular invasion (LVI) were found to have a significant impact on DFS as well as on OS. In the multivariate analysis only age (HR=1.79; 95%CI=1.14–2.82; p=0.012) and nodal status (HR=2.71; 95%CI=1.64–4.46; p<0.001) retained their independent prognostic value for DFS and for OS only the nodal status (HR=2.96; 95%CI=1.51–5.82; p=0.002).

**Conclusions:**

In our series of TNBC pts nodal status and age (older than 65 yrs) were found to be independent prognostic factors for DFS, whereas for OS only the nodal status. We found a pattern of a high recurrence rate in the first 3 years following the diagnosis and a decline in the recurrence rate over the next 3 yrs with higher rate of distal versus local recurrence and a predominant localization of distal metastases in visceral organs.

## Introduction

Breast cancer is the most common female cancer worldwide. It is a heterogeneous disease with regard to biological behaviour, responses to treatment and prognosis.^1^,^2^

Seventy to 80% of all breast cancers are positive for estrogene (ER) or progesterone receptors (PgR). In contrast, the human epidermal growth factor receptor (HER2) protein overexpression and/or HER2 gene are overexpressed and/or amplified, respectively, in approximately 15–20% of the patients only, with around half of these coexpressing hormone receptors. The remaining 10–15% of breast cancers is negative for ER, PgR and HER2. These are defined as triple negative breast cancer (TNBC).^3^

Among all the breast cancer subtypes, TNBC is associated with a worse prognosis. It has a characteristic recurrence pattern with the peak risk of recurrence and the majority of deaths occurring in the first 3 and 5 years after the initial treatment, respectively. Comparing to endocrine sensitive tumours, the risk for the late recurrence (beyond 5 years after the diagnosis) decreases by 50%.

Over the past decade, the landscape of breast cancer has changed. Steroid hormone receptors such as ER and PgR in concert with the HER2 still remain critical determinants of breast cancer subtypes and the treatment decision in daily clinical practice. But the development of microarray techniques evidenced inhomogeneous gene expression profiles and further divided breast cancer into several subtypes: luminal A, luminal B, HER2-enriched, and »basal-like« subtype. Both luminal A and B are clinically characterized by the expression of hormone receptor-related genes, whereas both HER2-enriched and the »basal-like« subtypes (BLBC) are less likely to express either ER or PgR. Moreover, the BLBC subtype is more commonly negative for all three markers. Subtypes vary in prognosis, with worse outcomes traditionally seen among the two hormone receptor negative subgroups compared to luminal subgroups.

Although frequently referred to interchangeably, the terms TNBC and »basal-like« are not completely synonymous. The term TNBC, namely, refers to the immunohistochemical classification of breast tumours lacking ER, PgR, and HER2 protein expression, whereas the »basal-like« subtype is defined via the gene expression microarray analysis. BLBC is, thus, characterized by the lack of expression of ER, PgR and HER2 (triple negative) as well as the increased expression of basal cytokeratins such as CK 5/6 and CK17. Although most BLBC do not express ER, PgR, HER2, a small number do and, therefore, the overlap between BLBC and TNBC is not complete. However, the triple negative phenotype currently serves as a reliable surrogate in the clinical practice.^4^ The heterogeneous nature of breast cancer has implications for physicians and their patients. Increasingly treatments are targeted toward molecular markers.

Because of the lack of expression of hormonal receptors and HER2, chemotherapy (ChT) remains the only systemic therapeutic option in the adjuvant and metastatic setting of this disease. Currently, no specific targeted approach is available for TNBC outside clinical trials.

The aim of our retrospective study was to analyse the clinicopathological characteristics and prognostic significance of putative prognostic factors in breast cancer as well as to determine short-and long-term outcomes in a group of consecutively treated patients with TNBC at the Institute of Oncology Ljubljana.

## Patients and methods

### Patients

In our retrospective analysis, we included 296 consecutively treated patients with TNBC treated from March 2000 until December 2006 at the Institute of Oncology Ljubljana. Patients with TNBC were identified from the database of the Department of Pathology. The established clinical and histomorphological factors such as menopausal status, pathological tumour size, tumour type, tumour grade, nodal status and hormonal receptor and HER-2 status as well as LVI were determined.

### Methods

We retrieved information on tumour characteristics from the pathology reports in the medical records of patients at the Institute of Oncology Ljubljana.

Tumour type was determined according to the UICC-WHO criteria and tumour grading was performed according to the Nottingham scheme.^5^ The steroid hormone receptor status was assessed by immunohistochemistry (IHC), using monoclonal rabbit ER antibody Clone SP1 (Neomarker) and monoclonal mouse anti-human PR antibody, Clone PgR 636 (Dako). Tumours were categorized as ER or PR positive if nuclear staining was observed in at least 10% of nuclei. The HER2 protein expression was determined by IHC using FDA approved HercepTest™ K5206 (DAKO) according to the recommended protocol. The membrane staining intensity and the pattern of the invasive component was evaluated according to Dako Cytomation’s ‘Atlas for Interpretation of HercepTest™’. Tumours were classified as IHC score 0 (negative) if no membrane staining or staining in less than 10% of the tumour cells was observed, an IHC score 1+ (negative) if a faint or barely perceptible partial membrane staining was detected in more than 10% of tumour cells, an IHC score 2+ (weakly positive) if weak or moderate complete membrane staining was observed in more than 10% of tumour cells and an IHC score 3+ (strongly positive) if complete strong membrane staining was observed in more than 10% of tumour cells.

The HER2 gene amplification was determined by dual-colour fluorescence *in situ* hybridisation (FISH) using FDA approved PathVysion® HER2 DNA probe kit and Paraffin pretreatment kit (both Abbot-Vysis). After whole slides were screened, HER2 gene and chromosome 17 centromere signals were counted in at least 20 nuclei and gene/ centromere ratio was calculated. If the ratio was borderline (between 1.7–2.3), signals were counted in additional 40 nuclei and ratio was calculated again. Tumours were classified as ‘not amplified’ (FISH−) if the calculated ratio was less than 2 and ‘amplified’ (FISH+) if the ratio was 2 or greater. The tumour was characterized as triple negative if hormone receptor status as well as HER2 status were both negative.

For urokinase plasminogen activator (uPA) and plasminogen activator inhibitor (PAI-1) determination, the tumour specimens were obtained by surgery and stored in liquid nitrogen until the extraction. The frozen tumour tissue samples were pulverized using a micro-dismembrator. The tumour powder was suspended in buffer (pH 8.5) containing 0.02 M Tris-HCl, 0.125 M NaCl and 2% Triton X-100 and shaken for 3 hours at 4ºC. The obtained suspension was then centrifuged for 30 min at 100000 × g. Protein content was determined according to the Pierce assay. Both biological markers were determined in tumour detergent extracts by commercially available enzyme-linked immunosorbent assays (American Diagnostica Inc., Greenwich, CT). Statistically optimized cutoff values were assigned for uPA (3 ng/mg protein) and PAI-1 (14 ng/mg protein).

Treatment decisions regarding the primary surgery and the adjuvant systemic therapy were based primarily on consensus recommendations at the time. After the completion of the primary treatment, patients underwent regular follow-up examinations at our institute.

All the procedures were in accordance with the ethical standards of our institute’s Ethical Committee.

### Statistical methods

The endpoints in this study were disease-free survival (DFS) and overall survival (OS). DFS was calculated from the date of the start of the primary therapy to the date of the breast cancer recurrence, the date of death from any cause, or the date of the last follow-up. OS was calculated from the date of the start of the primary therapy and death of any cause. DFS and OS as a function of the markers studied were estimated by the Kaplan-Meier method and the log-rank test was used to test for differences. The Cox multivariate hazards models were used to calculate the hazard ratios (HR) and their 95% confidence intervals (95% CI) in the analysis of DFS and OS. Computations were performed with the use of the SPSS 18 statistical package. The differences in the treatment between age groups were calculated using Pearson Chi-Square test. All reported p values are two tailed.

## Results

### Patients

At the time of the primary treatment, none of the patients had any evidence of distant metastases. The tumour’s, patient’s and treatment characteristics are presented in Table 1. The median age of the patients was 55 years (range, 23–88.5). The majority of women were postmenopausal at the presentation (60.3%).

Patients were more likely to have grade III tumours (82.5%), tumour size was larger than 2 cm in almost two thirds (59%). At least one axillary lymph node was positive in 46.1% of patients. One third of the tumours were positive for lymphovascular invasion (LVI). Of 185 patients with determined uPA and PAI-1 value, 141 had uPA ≥3 ng/ mg and 112 patients PAI-1 ≥ 14 ng/mg.

All the patients underwent the radical local treatment. Most of the patients (80%) were treated with some kind of ChT.

### Follow-up

The median follow-up was 5.9 years (range 0.3–9.6 years). Six (7.1%) patients experienced local, 79 (92%) patients distal recurrence and 66 (24%) died. After 5 years of follow-up the relapse developed only in 6 patients and only 4 died.

### Survival plots

**FIGURE 1.**
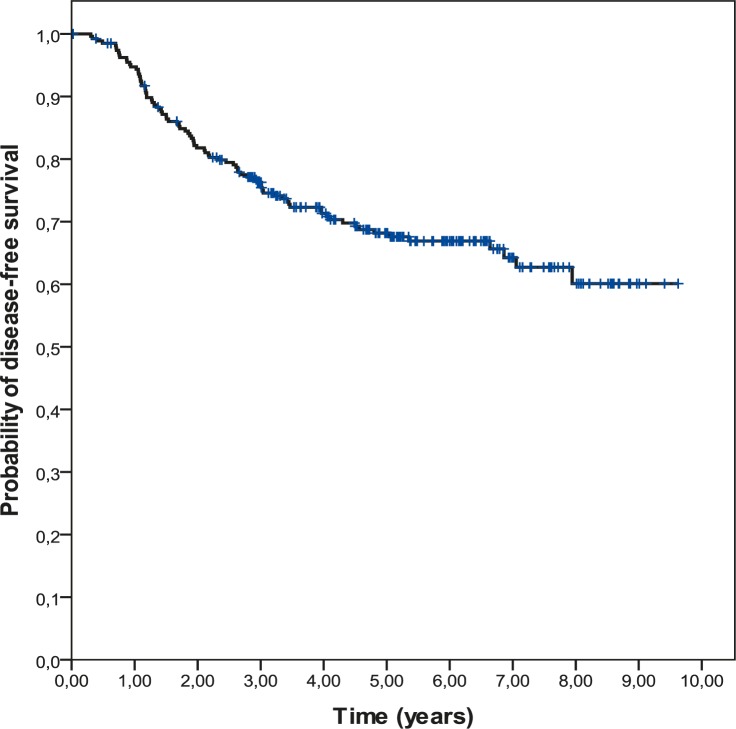
Disease-free survival (DFS) in 269 triple negative breast cancer (TNBC) patients.

**FIGURE 2.**
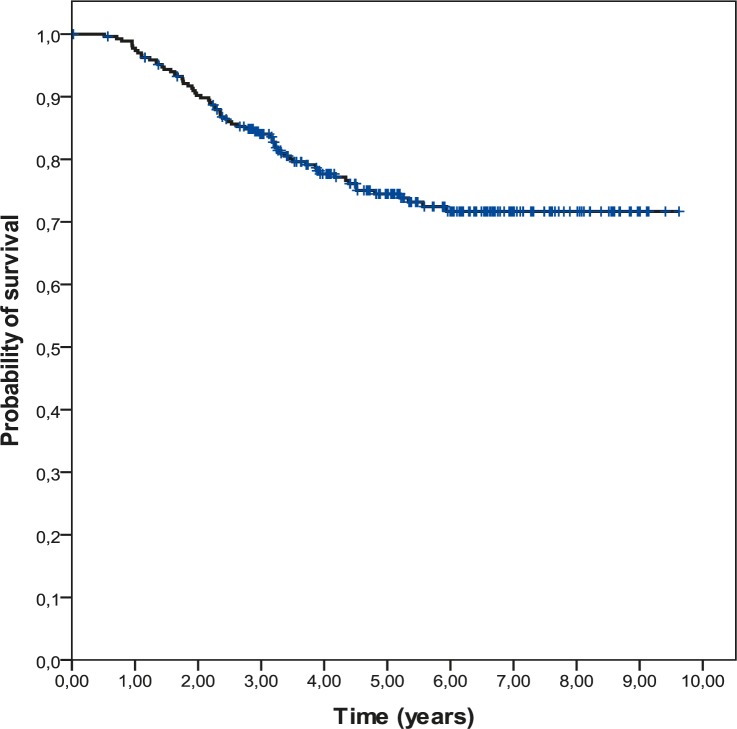
Overall survival (OS) in 269 triple negative breast cancer (TNBC) patients.

The 5-year DFS was 68.2% and the 5-year OS was 74.5%. Survival curves are shown in the Figures 1 and 2.

### Univariate and multivariate survival analysis

In the univariate analysis age, nodal status, size, and LVI were found to have significant impact on DFS as well as on OS while the menopausal status, tumour grade, uPA and PAI-2 had none.

In the multivariate analysis (Cox model) for DFS, age and nodal status retained its independent prognostic value. The patients with positive lymph nodes had a 2.71-fold higher risk of relapse (95%CI = 1.64–4.46). The risk of relapse was 1.79-fold higher in patients younger than 65 years compared with older patients (95%CI = 1.14–2.82). For OS only nodal status was an independent prognostic factor. The risk of death was 2.96 higher in patients with positive lymph nodes (95%CI = 1.51–5.82) (Table 2).

## Discussion

Emerging data on the clinical implication of the triple-negative phenotype indicate an aggressive course of this disease. Despite the widespread acknowledgment of the poor clinical outcome of TNBC, the prognostic value of specific morphological and biological features of these tumours continues to raise a substantial degree of uncertainty and controversy.

To date, studies on patients with TNBC have been limited mostly by the small sample sizes and short follow-up times. Our retrospective analysis was conducted in a relatively large number of consecutive patients (296) treated in the routine clinical practice with the median follow-up time of almost 6 years.

The majority of our TNBC patients had relatively large tumours at presentation (>2cm in 59% of patients), predominant type of tumour was invasive ductal carcinoma (90.7%), the majority of tumours were poorly differentiated (82.5%), almost half of patients had positive axillary lymph nodes at presentation (Table 1). Also in some previous reports triple-negative tumours were described as relatively large tumours (>2cm) with a high rate of node positivity.^1^,^3^,^5^ Similar to our study also other investigators found that characteristically TNBC exhibit an invasive ductal histology and a high histologic grade, present with high mitotic index, frequent apoptotic cells and carry central necrotic zones and pushing borders as well as a conspicuous lymphocytic infiltrate.^1^,^4^,^6^ In the population based Carolina Breast Cancer Study (CBCS), basal like breast cancers (defined by triple negative status plus EGFR or cytokeratin 5 positivity) were virtually all of ductal or mixed histology (90%), and of high grade (84%), which is similar to our results.^7^

In our analysis, the prognostic significance of putative well-known prognostic factors was assessed. We considered well-established prognostic factors such as menopausal status, age, nodal status, size of the tumour, grade, the presence of LVI, uPA, PAI-1, and type of adjuvant ChT. In the multivariate analysis only age and nodal status were found to be independent prognostic factors for DFS, whereas for OS only nodal status.

In patients older than 65 years the risk of relapse was 1.79-fold higher compared with younger patients (95%CI= 1.14–2.82, p=0.012). The explanation for this finding is probably in the difference in the treatment modality which is one of the most important prognostic factor in oncological patients.^8^ Due to the fact, that the elderly patients were treated with adjuvant ChT in a significantly smaller proportion compared to younger patients (46.8 vs. 91.1%, p< 0.001) such result was not surprising.

Since the nodal status is well established as one of the strongest prognostic factor in breast cancer, it was expected to show its prognostic value also in our study. The patients with positive lymph nodes had a 2.71-fold higher risk of relapse (95%CI= 1.64-4.46, p=0.002) and 2.96 higher risk of death (95%CI= 1.51–5.82, p<0.001) comparing to patients with negative axillary lymph nodes. These results are in line with some other studies.^1^,^7^,^9^ However, some other studies did not confirm the prognostic significance of the nodal status in TNBC^10^,^11^, therefore, the earlier detection^12^, which can improve OS in breast cancer patients, needs to be demonstrated.

Next to nodal status and age, tumour size and LVI were found as prognostic markers in the univariate analysis but lost the independency in multivariate analysis. In multiple recently published studies these tumour characteristics were demonstrated as important prognostic factors.^9^,^13^,^14^

The results from published literature showed that patients with TNBC have an increased likelihood of distant recurrence and of death compared to women with other types of breast cancer. The pattern of recurrence is also qualitatively different. In our analysis, we found a pattern of high recurrence rates in the first 3 years following the diagnosis and a clear decline in recurrence rate over the next 3 years (Figure 1). The study of Dent *et al*., which included large cohort of 1601 breast cancer patients demonstrated increased likelihood of distant recurrence (HR 2.6; 95%CI 2.0–3.5; p<0.0001) and death (HR 3.2; 95%CI 2.3–4.5; p<0.0001) within 5 years of diagnosis in the subgroup of 180 TNBC patients. On the contrary, among other, non-TNBC group, the recurrence risk was mostly constant over the period of the follow up.^13^ The study evaluating the response to neoadjuvant ChT among more than 1000 patients treated at the University of Texas M.D. Anderson Cancer Center corroborated the above prognostic findings. Results demonstrated decreased 3-years progression free and overall survival rates for triple-negative compared with non- triple-negative breast cancer. Consistently with previous reports, recurrences and death rates were higher only in the first 3 years following the diagnosis.^15^ The observed pattern speaks of the early aggressive nature of TNBC. Thus despite having a high risk of early recurrence, it seems that women with TNBC who are disease free after 5 years are unlikely to die of breast cancer.

Few women (7.1%) in our study cohort experienced a local before distal recurrence. This result is in line with some other studies.^9^,^13^,^14^ The high rate of distal recurrence and the relative rarity of local recurrences suggest that the mode of spread of these cancers is haematogenous and that these patients have a tendency to develop visceral metastases early in the course of their disease.

In addition to patterns observed in the timing of recurrence, the preferential site of relapse has also been identified among TNBC.^1^,^4^ Predominant localisation of the first relapse in our study were visceral organs (67.1%). Liedke *et al.* reported that TNBC patients have likewise higher rates of recurrence in visceral organs with lower rates of bone disease (74 vs. 13%, p=0.027), compared with hormone sensitive tumours.^14^ In the largest report to date, data on 12 858 patients, 2143 of them were triple negative, indicate on increased risk for lung and brain metastases as first site of recurrence and a lower risk for bone recurrence in patients with TNBC.^15^ Recent studies also indicate the increased incidence and uniquely aggressive nature of brain metastases in TNBC patients compared with other subtypes. Beside that diagnosis of central nervous spread is mostly followed by the shorter median survival of 3–5 versus 7–12 months in patients with TNBC compared with non-TNBC.^1^,^4^

It is not yet certain whether the poor prognosis of TNBC is due to the aggressive behaviour or because of the lack of the targeted therapy. The results from neoadjuvant and metastatic studies show that TNBC is relatively chemosensitive disease, with a good initial response to anthracycline and anthracycline/taxane ChT, but with a rapid relapse rate.^1^,^15^,^17^,^18^ In our cohort 80% of patients received adjuvant ChT. The majority of them were treated with anthracycline based ChT (60%), a quarter of patients received anthracyclines beside taxanes as well and only minority combination of cyclophosphamide, methotrexate and 5-fluoracil (CMF) (Table 1). We did not find a significant difference in outcome according to the treatment schedule.

To date novel therapeutic options are needed to target this aggressive type of breast cancer. Because of the lack of expression of hormonal receptors and HER2, ChT still remains the only possible systemic therapeutic option in the adjuvant and metastatic setting. There is currently no specific systemic regimen recommended for the treatment of TNBC and there is little data on which to base the treatment selection. Numerous efforts are currently being undertaken to improve prognosis for patients with TNBC. They comprise both optimization of choice and scheduling of common cytotoxic agents as well as the introduction of novel targeted agents. In terms of ChT DNA-damaging platinum chemotherapeutic agents are quickly emerging as the ChT »backbone« of choice in TNBC, especially when combined with novel agents such are poly ADP-ribose polymerase 1 (PARP1) inhibitors. Tumours with BRCA1 dysfunction, the majority of which are triple negative, namely harbour deficient double-stranded DNA break repair, which leads to increased sensitivity to these agents. The association between BRCA1 dysfunction and TNBC has led to several studies in metastatic and adjuvant/ neoadjuvant setting evaluating platinum agents in the setting of TNBC.^1^

As we are gaining a deeper understanding of the biology processes driving triple-negative breast cancer, the arena of targeted therapeutic agents is evolving. Potential targets for the treatment include: surface receptors such as epidermal growth factor receptor (EGFR), or c-Kit; protein kinase components of the mitogen activated protein (MAP)-kinase pathway; protein kinase components of the protein kinase B (Akt) pathway; induction of DNA damage by specific chemotherapy agents that cause interstrand and double-stranded breaks; and inhibition of already defective DNA repair by PARP1 inhibition.^6^ New knowledge on TNBC biology has, thus, revealed several promising targeted strategies, next to PARP1 inhibitors also EGFR-targeted agents (cetuximab), antiangiogenic agents (bevacizumab), inhibitors of Src-family kinases (dasatinib), histone deacetylase inhibitors and others, which are currently being tested in ongoing studies.^1^ One of the most exciting finding in the field of TNBC are definitely PARP-1 inhibitors. Results from two phase II clinical trial with two of them were presented in year 2009. A single arm trial of olaparib as single agent showed promising results in BRCA-deficient population.^19^ In randomised phase II study BSI-201 in combination with ChT with carboplatin and gemcitabine significantly improved overall and progression-free survival in women with metastatic TNBC, compared with ChT alone.^20^

## Conclusions

In conclusion, reviewing our data we were able to confirm that the TNBC is aggressive disease with a distinct pattern of recurrence. This pattern is characterized by a rapidly raising rate of recurrence within the first 3 years after the diagnosis and by a decline in a recurrence risk after 5 years from the diagnosis. Given that fact and the high risk of visceral metastases, these breast cancer patients may require closer surveillance in the initial years of the follow-up. However, the hypothesis that earlier detection and aggressive therapy of metastatic recurrence could improve survival needs to be demonstrated. Current results illustrate the need to develop novel therapeutic alternatives for this subgroup of patients.

## Figures and Tables

**TABLE 1.** Patient and tumour characteristics

**Characteristic**	**Number**	**% (of known)**
**Age (median, range)**	**55 (23–88.5)**	
**Menopausal status**		
pre/perimenopausal	104	39.7
postmenopausal	158	60.3
unknown	7	
**Tumour type**		
invasive ductal	244	90.7
invasive lobular	10	3.7
other invasive	15	5.6
**Size**		
≤ 2 cm	107	41.0
>2cm	154	59.0
unknown	8	
**LVI**		
no	189	75.3
yes	62	24.7
unknown	18	
**Grade**		
I	7	2.7
II	39	14.8
III	217	82.5
unknown	6	
**Nodal status**		
positive	123	46.1
negative	144	53.9
unknown	2	
**uPA**		
<3 ng/mg protein	44	23.8
≥ 3 ng/mg protein	141	76.2
unknown	84	
**PAI-1**		
<14 ng/mg protein	73	39.5
≥ 14 ng/mg protein	112	60.5
unknown	84	
**Chemotherapy regimen (adjuvant or neoadjuvant)**		
without chemotherapy	53	19.7
anthracycline based	129	48
CMF	31	11.5
anthracyclines and taxanes	53	19.7
other	3	1.1
**Localisation of first relapse (N=85)**		
local relapse only	6	7.1
visceral ± other localisations	57	67.1
soft tissues and bones	3	3.5
soft tissues only	7	8.2
bones only	12	14.1

LVI = lymphovascular invasion; uPA = urokinase plasminogen activator; PAI-1 = plasminogen activator inhibitor; CMF = cyclophosphamide, methotrexate and 5-fluoracil

**TABLE 2.** Univariate and multivariate analysis (Cox model) for 269 TNBC patients

**Characteristic**	**PFS**			**OS**		
	**univariate**	**multivariate**		**univariate**	**multivariate**	
	
	p	p	HR (95%CI)	p	p	HR (95%CI)
**Menopausal status** (pre/peri vs. postmenopausal)	0.172	-	-	0.278	-	-
**Age** (≥65yrs vs <65 yrs)	0.009	0.012	1.79 (1.14–2.82)	0.035	ns	-
**Nodal status** (positive vs. negative)	<0.001	<0.001	2.71 (1.64–4.46)	0.001	0.002	2.96 (1.51–5.82)
**Size** (>2cm vs. ≤ 2 cm)	0.004	ns	-	0.002	ns	-
**Grade** (III vs. I+II)	0.315	-	-	0.917	-	-
**LVI** (yes vs .no)	<0.001	ns	-	0.006	ns	-
**uPA** (≥ 3 ng/mg prot vs. <3)	0.827	-	-	0.732	-	-
**PAI-1** (≥ 14 ng/mg prot vs. <14)	0.487	-	-	0.632	-	-
**ChT regimen** (anthracycline based *vs*. anthracycline + taxanes *vs*. CMF)	0.234	-	-	0.071	-	-

LVI = lymphovascular invasion; uPA = urokinase plasminogen activator; PAI-1 = plasminogen activator inhibitor; ChT = chemotherapy; CMF = cyclophosphamide, methotrexate and 5-fluoracil

**TABLE 3.** Treatment differences according to age groups. Comparison of proportion of patients treated with adjuvant chemotherapy (ChT) according to age

**Age group**	**ChT yes (%)**	**ChT no (%)**
≥ 65 years (N=77)	41 (53.2)	36 (46.8)
< 65 years (N=192)	175 (91.1)	17 (8.9)

	p<0.001*	

* Pearson Chi-Square
